# Adaptation of the Upper Limb Functional Index (ULFI) to a Polish version and validation in patients with upper limb musculoskeletal disorders

**DOI:** 10.1186/s12891-025-08758-x

**Published:** 2025-05-23

**Authors:** Agnieszka Bejer, Jędrzej Płocki, Marek Kulczyk, Markus Melloh

**Affiliations:** 1 Faculty of Health Sciences and Psychology, Collegium Medicum, University of Rzeszow, Rejtana16C, Rzeszow, 35-959 Poland; 2 New Medical Techniques, Specialist Hospital of St. Family in Rudna Mała, Rudna Mała 600, Głogów Małopolski, 36-060 Poland; 3 Department of Physiotherapy, Collegium Medicum, University of Information Technology and Management in Rzeszow, Sucharskiego 2, Rzeszów, 35–225 Poland; 4 Institute of Public Health, School of Health Sciences, ZHAW Zurich University of Applied Sciences, Katharina-Sulzer-Platz 9, Winterthur, 8400 Switzerland

**Keywords:** Upper limb, Questionnaire, Psychometrics, Outcome measure

## Abstract

**Background:**

The Upper Limb Functional Index (ULFI) is a robust, widely used tool for assessing the functional status of the upper limbs (ULs) and the effectiveness of interventions in patients with musculoskeletal disorders (MSDs). This study aimed to translate and culturally adapt the ULFI into Polish (ULFI-PL) and evaluate its psychometric properties and practical characteristics.

**Methods:**

A total of 127 patients (54% female, $\overline{x }$ = 45.07 ± 14.97 years) with various ULMSDs and symptom durations completed the ULFI-PL, the shortened Disabilities of the Arm, Shoulder, and Hand questionnaire—Polish version (QuickDASH-PL), the Polish version of the World Health Organization Quality of Life—BREF (WHOQOL-BREF-PL), Numeric Pain Rating Scale (NPRS)*,* and the seven-point Global Rating of Change (GRC) scale*.* The internal consistency, construct validity, and factor structure were assessed in all the participants; the test–retest reliability and measurement error were evaluated in a subgroup (*n* = 112, 2–3-day interval); and the responsiveness and interpretability were evaluated in another subgroup (*n* = 56, 8-week interval, after physiotherapy).

**Results:**

The ULFI-PL demonstrated a good internal consistency (α = 0.77) and high construct validity, supported by the confirmation of five out of six a priori hypotheses (83.33%). A confirmatory factor analysis (CFA) revealed a unidimensional structure. It also demonstrated a high test–retest reliability (*r* = 0.85). The measurement error was calculated using the standard error of measurement (SEM = 4.75%) and the minimal detectable change (MDC_95_ = 13.17%). The ULFI-PL showed a high responsiveness after physiotherapy, which was confirmed by the effect size (ES = 2.08) and the standardized response mean (SRM = 1.88). The minimal clinically important difference (MCID) for the ULFI-PL was 28.29% (95% CI: 24.96–31.63).

**Conclusions:**

The ULFI-PL is a reliable, valid, and responsive tool for assessing the upper limb function in Polish-speaking patients with ULMSDs and is suitable for use in both clinical and research contexts. The results are consistent with previous studies on the original English version and other language adaptations.

## Background

In recent times, there has been a dynamic increase in the incidence of upper limb injuries and disorders, which tend to become more frequent with age [1]. These conditions are among the most burdensome, as they significantly hinder the performance of daily activities. The most significant results of impaired upper limb function include difficulties with personal hygiene, an inability to prepare meals, and limitations in social interactions [2]. Another important aspect of upper limb injuries is their impact on mental health, as they often lead to anxiety and stress. All of these factors can contribute to a reduced quality of life [3].

Patient-reported outcome measures (PROMs) can be categorized into generic and specific questionnaires and are used to assess patients' subjective perceptions of their health status [4]. Specific PROMs are developed using qualitative research methods, such as in-depth interviews and focus groups with patients affected by a given condition. This ensures that the questionnaire items are both relevant and acceptable to patients. Specific PROMs, compared to generic ones, exhibit greater responsiveness, making them more suitable for evaluating treatment outcomes. Musculoskeletal PROMs include both condition- and region-specific tools. Condition-specific PROMs assess symptoms related to a particular disease or injury, or a group of conditions that may affect a specific joint, as well as the resulting impairments in physical and mental functioning and health-related quality of life [5]. Numerous such questionnaires are available in the literature, as they focus on specific conditions. An example of a condition-specific PROM is the Western Ontario Rotator Cuff (WORC) questionnaire, which has a Polish version and is used to assess quality of life in patients with rotator cuff disorders [6, 7]. Another example is the Shoulder Pain and Disability Index (SPADI), designed to evaluate pain and disability associated with various disorders of the shoulder complex [8, 9]. More recently, region-specific PROMs have gained popularity due to their broader applicability in both clinical and research settings. These tools consider the body in single kinetic chains of the spine and upper and lower limbs, providing a clearer evaluation of clinical status and treatment-related changes. Compared to objective clinical measures, region-specific PROMs are more practical, easier to implement, and administer [10]. In the literature, only a few region-specific PROMs for the upper limb are available. Some of the most commonly used include the Upper Extremity Functional Scale (UEFS) [11], the Disabilities of the Arm, Shoulder, and Hand (DASH) questionnaire [12] and its shorter version, QuickDASH [13], as well as the Upper Limb Functional Index (ULFI) [10, 14]. The UEFS is a short eight-item questionnaire used to evaluate the influence of various upper limb disorders and the level of disease progression on the functioning of the working population [11]. The DASH and its shorter version—the QuickDASH, contain 30 and 11 items, respectively. They cover topics such as the severity of symptoms, difficulties in performing upper limb activities, and the impact of disability on social activities, sleep, or self-perception. They also include two optional modules for assessing the ability to perform work duties and participate in sports activities [12, 13]. The ULFI supports the “single kinetic chain” model of the upper limbs and has strong psychometric properties. The initial development and validation work was first published in 2006 (Gabel et al.) [10], and the tool was later modified in 2010 from a dichotomous to a three-point response option (“yes” = 1, “partly” = 0.5, or “no” = 0) to improve the reliability and reduce the cognitive load (Gabel et al.) [14]. The ULFI-3pt has a high internal consistency (Cronbach's α = 0.92), an excellent test–retest reliability (intraclass correlation: ICC2,1 = 0.98; minimal detectable change: MDC90 = 7.93%; standard error of measurement: SEM = 3.41%), a good concurrent validity with the QuickDASH (Pearson’s correlation coefficient: *r* = 0.86), a high responsiveness (effect size: ES = 0.93; standard response mean: SRM = 1.33), and a unidimensional factor structure. The ULFI has some advantages for clinicians and patients. Due to its very good practical characteristics, such as conciseness, understandability, and an ability to be quickly completed and scored, it has the potential for widespread use in clinical practice and research [10, 14]. There are also cross-cultural adaptations of the ULFI available in Spanish (ULFI-Sp, Cuesta-Vargas et al.) [15], French-Canadian (ULFI-FC, Hamasaki et al.) [16, 17], Turkish (ULFI-Tr, Tonga et al.) [18], Italian (ULFI-I, Sartorio et al.) [19], Korean (ULFI-K, In et al.) [20], Brazilian Portuguese (ULFI-Br, Takahasi et al.) [21], Persian (ULFI-Pr, Mokhtarinia et al.) [22], Urdu (ULFI-U, Arooj et al.) [23], Arabic (ULFI-Ar, Albahrani et al.) [24, 25], and Greek (ULFI-Gr, Chamogeorgakis et al.) [26], along with assessments of their psychometric properties. This suggests that the ULFI has great potential for further successful language adaptations, including into Polish.

In Poland, researchers and clinicians have access to many reliable and valid questionnaires specific to diseases/injuries or joints. However, there is a lack of PROMs that can be regionally used and consider the upper limb as a single kinetic chain. Currently, only the Polish versions of the DASH and QuickDASH questionnaires are available [27], which are recommended by the DASH Outcome Measure Institute for Work & Health (https://dash.iwh.on.ca.). However, these tools require further research regarding their psychometric properties and practical characteristics. Considering the above, this study was undertaken with the goal of translating and culturally adapting the ULFI for the Polish population, as well as assessing the psychometric properties and practical characteristics of the developed tool (ULFI-PL) among patients with various upper limb musculoskeletal disorders.

## Methods

### Participants

Consecutive patients who had appointments between January and December 2023 with a physiatrist or orthopedist at the New Medical Techniques, Specialist Hospital of St. Family in Rudna Mała, Poland, were prospectively recruited for this prospective cohort study, with inclusion criteria such as: having diseases/injuries located on the upper limbs; being assessed by a specialist through an interview, a physical examination, and imaging tests when needed (e.g., USG, MRI, CT, X-ray); having symptoms lasting longer than 1 week; being over 18 years old; being a native speaker of Polish; and having provided informed consent. The exclusion criteria included coexisting neurological diseases and an inability to read in Polish.

### Ethical approval

This study was conducted in accordance with the Declaration of Helsinki. The Bioethical Commission of the Rzeszow University granted permission to conduct the research (resolution No. 5/03/2021). Written informed consent was obtained from all the participants.

### Design

The evaluation followed a two-phase approach. The first phase involved translating the ULFI into Polish and culturally adapting it. The second phase focused on an evaluation of the developed Polish version of the questionnaire.

#### Phase 1: Translation and cultural adaptation of the ULFI to a Polish version

**Fig. 1:**
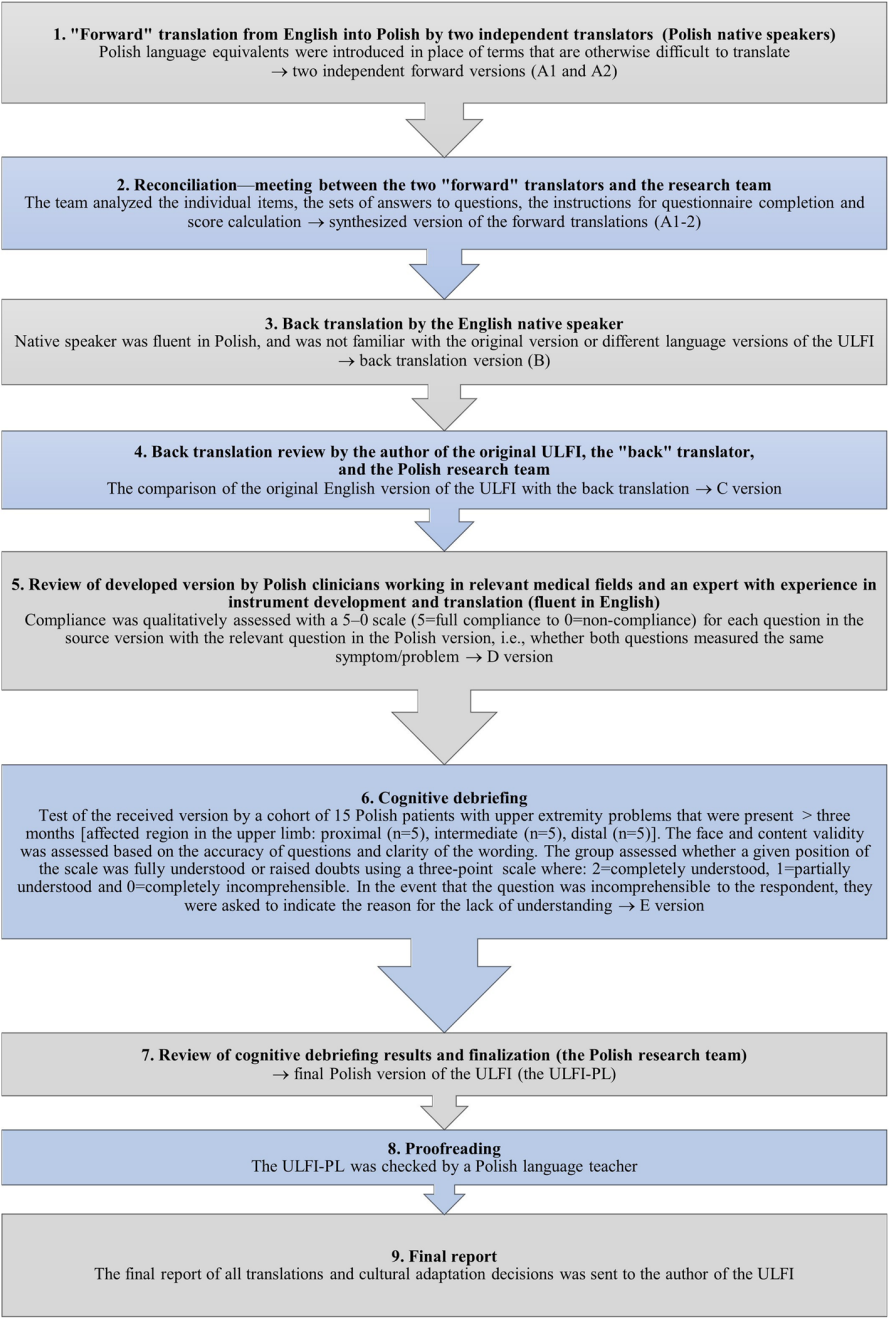
Flow chart of the translation and cultural adaptation process of the ULFI from English to Polish. *n*, number; *ULFI* Upper Limb Functional Index, *ULFI-PL* Upper Limb Functional Index—Polish version

The ULFI was translated and culturally adapted to a Polish version using the guidelines specified by the International Society for Pharmacoeconomics and Outcomes Research (ISPOR) and was approved by the Food and Drug Administration (FDA) and the European Medicines Agency (EMA) [28] (Fig. 1).

#### Phase 2: Prospective evaluation of the psychometric and practical properties of the ULFI-PL

### Data collection

The assessment of the participants was conducted three times. The baseline assessment (Test 1) utilized the ULFI-PL, the QuickDASH-PL, the WHOQOL-BREF-PL, and the NPRS. During the second assessment (Test 2; 48–72 h after Test 1, with no physiotherapy administered), the participants completed the ULFI-PL and a 7-point GRC. Test 3, which took place eight weeks after the baseline assessment, involved only those patients who received physiotherapy after Test 1 (3–6 weeks, depending on the recommendations of the physician/physiotherapist or the patient's condition). They completed the ULFI-PL, and the QuickDASH-PL and were also asked to report changes in their symptoms using a 7-point GRC.

### Research tools

#### The Upper Limb Functional Index, Polish version (ULFI-PL)

The ULFI-PL consists of 25 questions regarding difficulties in performing daily activities with the upper limbs. The responses are given on a 3-point scale, where 0 indicates no difficulty, ½ indicates partial difficulty, and 1 indicates difficulty in performing the given activity. The score is obtained by summing the points for each question and then multiplying this value by 4. The resulting number is then subtracted from 100 to obtain the final score in percentage terms. The scores vary from 0 to 100, with higher scores representing less upper limb functional impairment [10, 14].

#### The Polish version of the shortened Disabilities of the Arm, Shoulder, and Hand questionnaire— (QuickDASH-PL)

The QuickDASH-PL consists of 11 questions regarding the ability to perform activities with the upper limbs and the impact of musculoskeletal disorders of the upper limbs on work and social activities. Golicki et al. performed the translation and cultural adaptation of the QuickDASH into the official Polish version and confirmed its content validity [27]. QuickDASH-PL is recommended by the DASH Outcome Measure Institute for Work & Health (https://dash.iwh.on.ca). The responses are formatted on a 5-point Likert scale, where the lowest value (1) indicates no symptoms, and the highest value (5) indicates unbearable symptoms. The score is calculated by summing the selected numbers, dividing by the number of responses, subtracting 1, and multiplying by 25. The QuickDASH-PL score varies from 0 to 100, with higher scores representing greater functional limitations of the upper limbs [27] (https://dash.iwh.on.ca).

#### The Polish version of the World Health Organization Quality of Life—BREF (WHOQOL-BREF-PL)

The WHOQOL-BREF-PL is used for assessing the quality of life for research and clinical purposes in both patients and healthy individuals [29]. Jaracz et al. confirmed that the WHOQOL-BREF-PL is a valid and reliable questionnaire for the Polish population [30]. It contains 26 questions grouped into four domains: physical, psychological, social, and environmental. Each question score ranges from 1 to 5. To determine the domain scores, the arithmetic mean of the items in each domain is calculated. They are positively oriented, meaning that the higher the number of points collected, the better the quality of life. [29, 30].

#### A 7-point Global Rating of Change (GRC) scale

The GRC scale consists of a single question in which the patients were asked to assess changes in upper limb function related to a specific condition over two defined periods: between the first and second examinations (with no physiotherapy) and between the second and third examinations (after physiotherapy). The responses were given on a 7-point Likert scale, with the midpoint (4) denoting “no change,” the lowest value (1) denoting “much better,” and the highest value (7) denoting “much worse” [31].

#### The Numeric Pain Rating Scale (NPRS)

The NPRS is an 11-point numerical scale in which patients are asked to select a whole number between “0” (no pain) and “10” (the worst pain imaginable) to represent their pain intensity experienced over the past few days. Patients were instructed to indicate pain intensity only for the upper limb(s) and not for other body regions [32].

### Statistical analyses

The analyses were computed using the R statistical package, version 4.2.2 [33]. A statistical significance level of *p* = 0.05 was set prior to the analysis. The sample’s descriptive statistics were summarized with the mean, standard deviation, median, and quartiles. To compare the changes between the 1st and the 3rd evaluation, Wilcoxon’s test for paired samples was used. It was assumed that floor and ceiling effects were present if the percentage of the lowest or highest scores was greater than 15% [34].

### Reliability analyses

#### Internal consistency

The internal consistency reflects the extent of inter-relatedness among the items. It was assessed using Cronbach`s alpha (α) coefficient (*n* = 127), where α is expected to fall between 0.70 and < 0.95 [34]. The item-total correlations (i.e., the correlation between an individual item score and the overall assessment score) are expected to be ≥ 0.3."

#### Test–retest reliability

The intraclass correlation (ICC_2.1_, CI = 95%) was used to assess the test–retest reliability. It refers to the capacity of a questionnaire to generate similar scores during two different testing instances, given that the patient's condition is unchanged. The data from patients who completed the ULFI-PL questionnaire twice within 48–72 h with a stable status between the test and the retest were used (*n* = 112). The test–retest reliability was considered good if the ICC was r ≥ 0.70 [35].

#### Measurement error

To assess the measurement error, the standard error of measurement (SEM) and the minimal detectable change at the 95% level (MDC_95_) were used (*n* = 112). The SEM denotes the variability in an individual’s scores from several administrations of the same test, and the larger the SEM, the greater the score variation across administrations. This was determined using the following formula: SEM = [SD√(1-R)], where SD = standard deviation of the measurement and R = test–retest reliability coefficient. The MDC estimates the minimal score alteration required to confirm that the change is genuine, beyond the measurement error. The MDC_95_ was computed using the relevant z-value [MDC_95_ = SEMx1.96x√2] [36].

### Validity analyses

#### Construct validity

The construct validity indicates the degree to which scores on a particular questionnaire correlate with other measures in a way that aligns with previously established theoretical hypotheses regarding the concepts being evaluated [34]. Therefore, to evaluate the construct validity, Spearman's rank correlation coefficient (r) was used with a 95% confidence interval (*n* = 127), and six a priori hypotheses were proposed as follows: The ULFI-PL should correlate highly with the QuickDASH-PL, which is also used to assess the function of upper extremities (the first hypothesis). It should correlate moderately (physical domain) or poorly (psychological, social, and environmental domains) with the WHOQOL-BREF-PL, because that is the generic questionnaire to assess quality of life (the second to fifth hypotheses). The ULFI-PL should also correlate moderately with the P-NRS, as it assesses only the severity of pain (the sixth hypothesis). The indications for the r strength for validity were < 0.30 = low, 0.30–0.70 = moderate, and ≥ 0.70 = high, and they should be statistically sufficient [37]. If fewer than 25% of the hypotheses were rejected, the construct validity of the ULFI-PL was deemed to be high. Moderate validity was indicated by rejecting 25–50% of the hypotheses, while low validity was suggested when > 50% were rejected [34].

#### Factor structure

A confirmatory factor analysis (CFA) was conducted (*n* = 127) to determine if the ULFI-PL maintained a unidimensional structure similar to the original questionnaire. The CFA utilized a polychoric matrix along with the robust diagonally weighted least squares (RDWLS) extraction method (factor loadings > 0.40) [38]. The model fit was evaluated using several indices: the chi-squared test (χ^2^), the root mean square error of approximation (RMSEA), the comparative fit index (CFI), the Tucker–Lewis index (TLI), and the standardized root mean square residual (SRMR). Following the criteria set by Hu and Bentler, the model was regarded as having a good fit (confirming the unidimensional structure of the ULFI-PL) if the indices met the following thresholds: RMSEA < 0.06, CFI and TLI > 0.95, SRMR < 0.08, and if the χ^2^ test yielded *p* > 0.05 [39].

### Responsiveness analyses

#### The standard effect size (ES) and standardized response mean (SRM)—internal responsiveness

The ES value was defined to correspond to a change in the ULFI-PL score (Tests 1–3; *n* = 56) divided by the baseline standard deviation (SD). The SRM value was calculated from the mean change in the score divided by the SD of the above change in the score. Absolute values of 0.20 or lower, between 0.21 and 0.79, and 0.80 or higher were considered to indicate a low, moderate, and high responsiveness, respectively—the capacity of a questionnaire to recognize clinically relevant changes over time—for the ES and SRM [34, 40].

#### Changes in the ULFI-PL and QuickDASH-PL—external responsiveness

We compared changes in the ULFI-PL and QuickDASH-PL scores between the baseline data and the eight-week follow-up evaluation using Spearman’s rank correlation coefficient (r) [37]. We hypothesized that changes in the ULFI-PL would be moderately to strongly correlated with changes in the QuickDASH-PL and would exhibit a negative correlation.

### Interpretability analysis

#### The minimal clinically important difference (MCID)

The MCID represents the smallest improvement that is perceived as beneficial by both patients and clinicians. The distribution-based method was applied to determine the MCID as the SEM for the difference in the ULFI-PL score (Test 1–3) in the patients "without change" group based on a 7-point GRC scale (4, 5 or 6 points on the GRC) [41].

### Practical characteristics

A qualitative readability analysis was included in the assessment of the face and content validity during the first phase (*n* = 15). The completed ULFI-PL used during the baseline examinations (*n* = 127) was checked for possible missing responses. The completion and scoring times were calculated from the measures of 15 patients that were performed by two physiotherapists.

### Sample size

The sample size was determined in accordance with the COSMIN recommendations, which state that, for a CFA, an adequate sample size should be a minimum of five times the number of items in the questionnaire, with a total sample size of at least 100 participants (for the ULFI, this would mean 125 patients). For the other analyses, a minimum of 50 patients is needed [42]. Additionally, a post-hoc analysis of test power was performed, considering the ICC. For the null hypothesis ICC = 0.7, with a sample size of 112 participants (individuals assessed in Test 1 and Test 2), a significance level of 0.05, and the anticipated ICC value in our study group, the result showed very high test power, exceeding 0.999 for the total score in the ULFI-PL. The sample size was found to be satisfactory in terms of statistical analysis.

## Results

### Phase 1: The ULFI translation and cultural adaptation to a Polish version

As a result of translating and culturally adapting the ULFI to Polish, several modifications were made throughout the process, as outlined below:

- Step 2. During the reconciliation meeting, several acceptable variations between the two forward translations were recognized, stemming from the various Polish language equivalents selected by the translators. A unified version was agreed upon.
- Step 5. The review carried out by bilingual practitioners from applicable medical fields highlighted four questions based on the panel’s feedback, leading to the following subsequent corrections:
  - #3 and #7: The weight provided in pounds was changed to the units used in Poland—kilograms.
  - #12: In the item “I need assistance with personal care, e.g., washing and hygiene,” the scope of personal care was clarified to focus on body care, in line with the examples provided. The item was corrected to “I need help with body care, e.g., washing and maintaining hygiene.”
  - #20: The examples of utensils used for eating were modified—"chopsticks,” which are not commonly used in Poland, were replaced with “spoon,” which is frequently used.
  - #25: Two examples among the items that patients may frequently “open, hold, push, or press” were changed—“triggers, levers” were modified to “flush handles, door handles.”
- Step 6. The cognitive debriefing, which analyzed the responses from fifteen symptomatic participants, indicated patient cognitive acceptance (average = 2.0/2.0), and no corrections were necessary.

The remaining steps also did not introduce any changes to the questionnaire. This translation and cultural adaptation process resulted in a Polish version of the ULFI (ULFI-PL).

### Phase 2: Prospective assessment of the psychometric and practical properties of the ULFI-PL study participant characteristics

Table 1 provides a detailed overview of the sociodemographic and clinical characteristics of the participants (*n* = 127, Test 1). In the second assessment, aimed at evaluating the stability of the ULFI-PL (2–3 days after the first test), 112 patients who had not received treatment participated (2 individuals declined to participate in the study, and 13 started physiotherapy). The third assessment (8 weeks after the first test), aimed at evaluating the responsiveness of the ULFI-PL, was conducted only for individuals who had undergone 3 to 6 weeks of physiotherapy (*n* = 56). The remaining patients did not participate in the follow-up: 34 were waiting for physiotherapy under health insurance, 19 were undergoing physiotherapy, 2 had undergone surgery, 6 were awaiting surgery, and 10 declined to participate in the study.

**Table 1:** Sociodemographic and clinical characteristics of the study population

Parameter		Total (*n* = 127)
Age [years]	$\overline{x}$** ± **SD	45.07 (14.97)
	Me (quartiles)	47 (32.75–57)
	Range	20–72
Sex	Female N (%)	68 (53.54%)
	Male N (%)	59 (46.46%)
Place of residence	Urban area N (%)	65 (51.18%)
	Rural area N (%)	62 (48.82%)
Education	University degree N (%)	36 (28.35%)
	Secondary N (%)	57 (44.88%)
	Vocational N (%)	31 (24.41%)
	Primary N (%)	3 (2.36%)
Professional activity	Professionally active N (%)	94 (74.02%)
	Student N (%)	15 (11.81%)
	Retired N (%)	12 (9.45%)
	Pensioner N (%)	6 (4.72%)
Limb with injury	Right N (%)	82 (64.57%)
	Left N (%)	45 (35.43%)
Dominant limb	Right N (%)	107 (84.25%)
	Left N (%)	20 (15.75%)
Time since injury/first symptoms [weeks]	$\overline{x}$** ± **SD	21.76 (34.09)
	Me (quartiles)	10 (5–20)
	Range	2–108
**Area**	**Diagnosis**	**n (%)**^**a**^
Shoulder	Rotator cuff injuries—treated conservatively/surgically	33 (29.47%)
	Shoulder joint instability—treated conservatively/surgically	7 (6.25%)
	Shoulder joint arthroplasty	1 (0.89%)
	Degenerative changes to the shoulder joint	11 (9.82%)
	Frozen shoulder	6 (5.36%)
	Subacromial impingement syndrome	13 (11.61%)
	Status post-humeral fracture—treated surgically	6 (5.36%)
Elbow	Tennis elbow	10 (8.93%)
	Golfer's elbow	5 (4.46%)
	Status post-ulnar/radial fracture—treated surgically/conservatively	10 (8.93%)
Wrist/hand	Carpal tunnel syndrome—treated surgically/conservatively	15 (13.39%)
	Status post-fracture/injury of the wrist/hand—treated surgically or conservatively	14 (12.50%)
	De Quervain's syndrome	7 (6.25%)
	Dupuytren's contracture	1 (0.89%)

*n*, number; *%*, percent; $\overline{x}$, mean; *SD*, standard deviation; *Me*, median

^a^Multiple choice question—percents do not sum up to 100

### The research tool absolute values

**Fig. 2:**
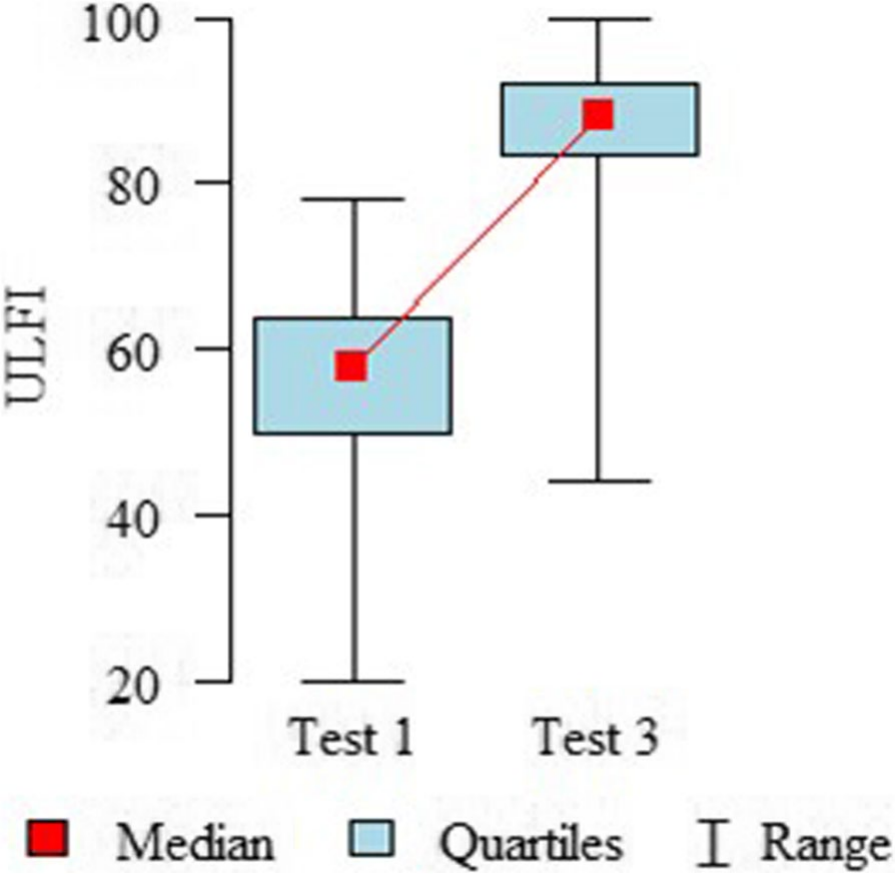
Distribution of the ULFI-PL Scores in Test 1 and Test 3. *ULFI* Upper Limb Functional Index

Table 2 and Fig. 2 provide the mean score, standard deviation, and score range achieved for each questionnaire. There were no floor or ceiling effects in the ULFI-PL questionnaire, because the percentage of the lowest or highest scores was less than 15%. Additionally, none of the participants achieved the minimum score on the ULFI-PL assessments, and the maximum score on the ULFI-PL was reached by fewer than 2% of participants in the third assessment only.

**Table 2:** Absolute values of all scores

Questionnaire		Test 1 (*n* = 127)				Test 2 (*n* = 112)				Test 3 (*n* = 56)			
		$\overline{x}$** ± SD**	**Range**	**Floor Score %**	**Ceiling Score %**	$\overline{x}$** ± SD**	**Range**	**Floor Score %**	**Ceiling Score %**	$\overline{x}$** ± SD**	**Range**	**Floor Score %**	**Ceiling Score %**
**ULFI-PL**		55.93 ± 12.23	20 – 78	0.00%	0.00%	57.27 ± 12.58	18—78	0.00%	0.00%	83.25 ± 13.12	44—100	0.00%	1.79%
**QuickDASH-PL**		34.15 ± 18.7	0—72.73	0.89%	0.00%	–-	–-	–-	–-	18.34 ± 16.45	0—61.36	12.50%	0.00%
**Domain**													
**WHOQoL-BREF-PL**	Physical	70.28 ± 19.36	31 – 100	0.00%	6.25%	–-	–-	–-	–-	–-	–-	0.00%	14.29%
	Psychological	68.82 ± 19.39	0 – 100	0.89%	5.36%	–-	–-	–-	–-	–-	–-	0.00%	16.07%
	Social	76.58 ± 20.96	19 – 100	0.00%	22.32%	–-	–-	–-	–-	–-	–-	0.00%	30.36%
	Environmental	74.48 ± 15.81	38 – 100	0.00%	9.82%	–-	–-	–-	–-	–-	–-	0.00%	19.64%
**NPRS**		4.66 ± 2.1	0 – 8	2.68%	0.00%	–-	–-	–-	–-	–-	–-	0.00%	0.00%

*n* number, $\overline{x}$ mean, *SD* standard deviation, *Me* median, *%* percent, *ULFI-PL* upper limb functional index—Polish version, *QuickDASH*-*PL shortened,* Disabilities of the Arm, Shoulder and Hand questionnaire—Polish version, *WHOQoL-BREF-PL* Polish version of the World Health Organization Quality of life—BREFF, *NPRS* Numeric Pain Rating Scale

### Reliability analysis

The internal consistency of the ULFI-PL was satisfactory, with α = 0.765 (Table 3), and item-total correlations ranging from 0.304 to 0.573 (Table 4). The test–retest reliability, assessed using the ICC_2,1_, was high, and amounted to 0.849, with a 95% confidence interval of 0.787–0.894 (*p* = 0.037). The measurement error was determined from the SEM and MDC_95_, at 4.75% and 13.17%, respectively (Table 3).

**Table 3:** Data from the reliability analysis and measurement error (*n* = 112)

	**Cronbach’s alpha (*n* = 127)**	**ICC (95%CI)**	**P**	**SEM**	**SEM%**	**MDC (95%CI)**	**MDC% (95%CI)**
ULFI-PL	0.765	0.849 (0.787—0.894)	0.037 *	4,752	8,241	13.172 (15.644—11.038)	21.21 (18.092—24.628)

*ULFI-PL*, upper limb functional index—Polish version; *ICC,* intraclass correlation; *SEM,* standard error of measurement; *MDC,* minimal detectable change; *CI,* confidence interval

^*^statistically significant (*p* < 0.05)

**Table 4:** Item-Total correlations (*n* = 127)

Items of the ULFI-PL	Item-Total correlations
1. Stay at home most of the time	0.423
2. Change positions frequently	0.306
3. Avoid heavy jobs	0.323
4. Rest more often	0.534
5. Get others to do things	0.409
6. The pain almost all the time	0.325
7. Lifting and carrying	0.37
8. Appetite affected	0.304
9. Walking/normal recreation/sport	0.405
10. Home/family duties and chores	0.505
11. Sleep less well	0.312
12. Assistance with personal care, hygiene	0.421
13. Regular daily activity work/social	0.373
14. More irritable/bad-tempered	0.474
15. Feel weaker or stiffer	0.327
16. Transport independence	0.492
17. Arm in shirt sleeve/dressing	0.45
18. Writing/using keyboard or mouse	0.419
19. Do things at/above shoulder	0.407
20. Eating: using utensils	0.326
21. Hold or move dense objects	0.458
22. Drop things—minor accidents	0.361
23. Use another arm more often	0.573
24. Difficulties—button / key / coins / taps	0.429
25. Open / hold / press / push	0.538

*ULFI-PL* upper limb functional index—Polish version

### Validity analysis

The construct validity was assessed based on the relationship between the ULFI-PL and the QuickDASH-PL (*r* = − 0.79, *p* < 0.001), and it indicated a strong and significant correlation. Moderate correlations were observed between the ULFI-PL and the NRS (*r* = -0.56, *p* < 0.001), as well as the physical domain of the WHOQoL-BREF-PL (*r* = 0.511, *p* = 0.001). Additionally, the ULFI-PL showed weak, but significant, correlations with the domains of the WHOQoL-BREF-PL that have less convergent constructs (psychological and social domains), as well as weak and non-significant correlations with its environmental domain. The confirmation of five out of the six a priori hypotheses (83.33%) reflects a high construct validity (Table 5).

**Table 5:** Correlations (r) between the ULFI-PL and the reference questionnaires (*n* = 127)

Questionnaire		ULFI-PL	
		**Spearman’s correlation coefficient (r) (95%CI)**	**p**
**QuickDASH-PL**		*r* = -0.794, (− 0.850, − 0.719)	*p* = 0.001*
	Physical domain	*r* = 0.511, (0.369, 0.629)	*p* < 0.001*
**WHOQoL**	Psychological domain	*r* = 0.278, (0.109, 0.431)	*p* = 0.041*
**- BREF-PL**	Social domain	*r* = 0.222, (0.050, 0.381)	*p* = 0.021*
	Environmental domain	*r* = 0.166, (− 0.009, 0.329)	*p* = 0.08
**NPRS**		*r* = -0.566, (− 0.674, − 0.435)	*p* < 0.001*

*ULFI-PL* Upper Limb Functional Index—Polish version, *QuickDASH-PL* shortened Disabilities of the Arm, Shoulder and Hand questionnaire—Polish version, *WHOQoL-BREF-PL* Polish version of the World Health Organization Quality of Life—BREFF, *NPRS* Numeric Pain Rating Scale, *95%CI* 95% confidence interval

*r* ≤ 0.30 = weak, 0.3 < *r* < 0.7 = moderate, *r* > 0.70 = strong correlation

^*^statistically significant (*p* < 0.05)

The structural validity fulfilled the a priori criteria. The CFA revealed a one-dimensional structure for the ULFI-PL, with appropriate factor loadings (> 0.40; *p* < 0.05) for all items, ranging from 0.381 (item 8) to 0.724 (item 23), as well as good fit-indices. The χ^2^ test indicated no statistical significance (*p* > 0.05), and the fit indices were found to be excellent according to the criteria proposed by Hu and Bentler [36] (RMSEA < 0.06, CFI and TLI > 0.95). Additionally, the SRMR was very close to the expected value (< 0.08) (Table 6).

**Table 6:** Data from the confirmatory factor analysis of the ULFI-PL (*n* = 127)

Chi-squared test			RMSEA	CFI	TLI	SRMR
**χ**^**2**^	**df**	p*				
245.661	251	0.583	0.00000026	0.99936	0.999352	0.089

***χ***^***2***^ chi-squared*, df* degrees of freedom,* p* statistical significance*, RMSEA* root mean square error of approximation, *CFI* comparative fit index, *TLI* Tucker–Lewis index, *SRMR* standardized root mean square residual

^*^ statistically significant (*p* < 0.05)

### Responsiveness analysis

#### Internal responsiveness

The calculated ES was 2.08, and the SRM was 1.88; thus, a large degree of responsiveness was shown.

#### External responsiveness

There was a significant change in the ULFI-PL between Test 1 and Test 3 (*n* = 56; $\overline{\text{x} }$ = 24.46; Me = 28; SD = 13.56; *p* < 0.001). The results increased, so a significant improvement in the functionality of the upper limbs was achieved. As predicted, a moderate and statistically significant correlation (*r* = -0.446, *p* = 0.009) was observed between the changes in the ULFI-PL and the changes in the QuickDASH-PL.

### Interpretability analysis

The MCID was calculated between Tests 1 and 3, and based on the distribution-based distribution method, it was 10.41 (95% CI: 7.62—16.41) and 13.06% (95% CI:9.52—19.75).

### Practical characteristics

No missing responses were detected in the completed ULFI-PL. The average completion time for the ULFI-PL was 144 ± 40 s, while the average scoring time was 18 ± 6 s.

## Discussion

This study involved the translation and cultural adaptation of a Polish version of the ULFI, as well as an investigation into its psychometric properties. The resulting ULFI-PL version demonstrated an adequate internal consistency, test–retest reliability, and construct validity. Additionally, the tool exhibited a unidimensional structure and was responsive to changes in the patients' status.

### Translation and cultural adaptation of the ULFI to a polish version

The cultural adaptation process did not reveal any major difficulties, which is consistent with other translation and adaptation studies on the ULFI [15–26]. Patients with injuries or diseases of the upper limbs who were familiarized with the questionnaire items and response options indicated that they were relevant and fully understandable. The final version of the ULFI-PL was completed by 127 patients with upper limb musculoskeletal diseases (ULMSDs) or injuries, or those who had undergone orthopedic surgery. The affected areas included the proximal (55.4%), middle (18%), and distal (26.6%) parts. This is similar to some previous studies (Cuesta-Vargas and Gabel, 2013; Tonga et al., 2015; In et al., 2017), which also showed a predominance of the proximal region (49%-76%) [15, 18, 20]. Additionally, 9% of the patients had two or more affected upper limb regions, highlighting the versatility of a region-specific instrument such as the ULFI for evaluating these patients.

### Prospective assessment of the psychometric and practical properties of the ULFI-PL

The internal consistency of the ULFI-PL was satisfactory, with α = 0.765, indicating that all the items were correlated and assessed the same construct. It was also below the threshold value of α = 0.95, indicating an item redundancy. However, the internal consistency of the ULFI-PL had slightly lower values compared to the original (α = 0.92) [14], as well as other language versions of the ULFI—Turkish and Arabic (α = 0.88) [18, 24], Italian and Brazilian Portuguese (α = 0.90) [19, 21], Persian (α = 0.91) [22], French-Canadian (α = 0.93) [16], Spanish (α = 0.94) [15], and Korean (α = 0.94) [20]. Moreover, all item-total correlations were ≥ 0.3, indicating that none of the ULFI-PL items should be removed. The test–retest reliability of the ULFI-PL was high, and the ICC amounted to 0.85 (0.787—0.894), which proves the stability of the instrument in repeated measurements. The ICC for the original questionnaire was 0.96 [14], while for other language versions, it ranged from 0.72 (Turkish) [18] to 0.97 (French-Canadian and Greek) [17, 26]. The measurement error was determined from the SEM and MDC_95_, which were 4.75% and 13.17%, respectively. These values were lower than those obtained for the Brazilian Portuguese version (SEM = 6.11%, MDC90 = 14.26%) [21] and comparable to those of the French-Canadian (SEM = 4%, MDC90 = 9.3%) [17], Arabic (SEM = 4.43%, MDC90 = 10.34%) [25], and Italian (SEM = 5%, MDC90 = 12%) [19] versions. Notably, lower measurement error values were obtained for the original ULFI (SEM = 3.41%, MDC_90_ = 7.93%) [14] and for the Turkish [18], Persian [22], Greek [26], Spanish [15], and Urdu [23] versions (SEM from 2.95% to 3.89%, MDC_90_ from 5.53% to 10.6%). The higher measurement error values observed in this validation of the ULFI may be related to the inclusion of a patient population with conditions ranging from acute to chronic (2 to 108 weeks since injury or the onset of initial disease symptoms). The SD value at baseline can increase considerably when the population has a wide variety of functional statuses. In the present study, the MDC_95_ was derived from the SEM, which in turn depends on the baseline SD value.

The ULFI-PL was shown to be a valid instrument for measuring upper limb functional impairment. In this study, 83.33% of the six a priori hypotheses were confirmed, which indicates a high construct validity according to the criteria proposed by Terwee et al. [34]. As hypothesized, the relationships between the ULFI-PL and another tool assessing the upper limb functional capacity, the QuickDASH-PL, were significant and strong (*r* = − 0.79). Most previous studies have demonstrated that the ULFI shows a strong correlation with the QuickDASH (ULFI-U: *r* = 0.86 [23], ULFI-original: *r* = 0.85 [14], ULFI-Gr: *r* = 0.75 [26]) and DASH (ULFI-FC: *r* = 0.85 [17], ULFI-K: *r* = 0.72 [20], ULFI-Pr: *r* = 0.71 [22], ULFI-Ar: *r* = -0.80 [25], as also reported in this study. The NPRS assesses pain that affects the functionality of the upper limbs; it shows moderate and significant correlations (*r* = -0.57) with the ULFI-PL, and also with other validations: (*r* = − 0.53) with the ULFI-Br [21], (*r* = -0.52) with the ULFI-U [23], (*r* = -0.50) with ULFI-Ar [25], and (*r* = -0.57) with the ULFI-Gr [26]. The ULFI-PL showed weak correlations with the domains of the WHOQoL-BREF-PL with less convergent constructs (environmental, social, and psychological domains (from *r* = 0.17 to 0.28) and moderate correlations with the physical domain (*r* = 0.51). Such correlations with the WHOQoL-BREF-PL could be expected, as it is a quality-of-life assessment instrument, not a region-specific tool such as the ULFI-PL. This is also supported by findings from other researchers. The ULFI-U version showed correlations with the SF-12 ranging from low (*r* = 0.09, vitality domain) to strong (*r* = 0.77, physical functioning domain) [23]. The Spanish version of the ULFI showed a moderate correlation (*r* = -0.59) with the EuroQol Health Questionnaire—five Dimensions (EQ-5D-3L) [15]. A similar correlation was also observed for the Brazilian Portuguese version between the ULFI-Br and the SF-36 (ranging from *r* = 0.31 for the mental health domain to *r* = 0.57 for the physical role domain) [21].

According to the concept of the ULFI authors, the questionnaire was designed to assess the functional status of the upper limbs as a single kinetic chain, providing a single summated score [14]. In this study, the CFA revealed a unidimensional structure of the ULFI-PL with appropriate factor loadings from 0.381 to 0.724 (*p* < 0.05) and with fit-indices [χ^2^ = 245.661, df = 251 (*p* = 0.583); RMSEA < 0.001; CFI > 0.999; TLI > 0.999; SRMR = 0.089] according to the conventional criteria proposed by Hu and Bentler [39]. According to these criteria, our computed fit index, SRMR was slightly above the expected threshold (< 0.08). However, these same authors note that meeting the criteria for all indices is often an overly strict requirement and propose the Two-Index Strategy, which assumes that the model is well fitted when SRMR < 0.09 and at least one of the following conditions is met: CFI > 0.96, TLI > 0.96, or RMSEA < 0.06 [39]. The fit models obtained in the CFA were also acceptable for the Brazilian [χ^2^ = 441.860, df = 252, RMSEA = 0.063, CFI = 0.918, TLI = 0.910] [21] and Arabic [df = 275, χ^2^ = 588.98 (*p* < 0.001), CFI = 0.652, RMSEA = 0.091, TLI = 0.620] versions [25]. The same unidimensional structure was also found using an exploratory factor analysis (EFA) on the Spanish [15] and Persian [22] versions. However, the Turkish [18], Greek [26], and Urdu [23] versions of the ULFI revealed a bidimensional structure; the Italian version demonstrated a multi-factor structure [19]; and the French-Canadian and Korean versions did not report any factor analysis results [17, 20].

Responsiveness refers to an instrument's ability to detect clinically significant changes resulting from an intervention [40]. The ULFI-PL demonstrated a large degree of responsiveness, as measured by the ES = 2.08 and the SRM = 1.88. According to Cohen’s criteria, these findings reinforce their suitability for tracking recovery progress and evaluating treatment outcomes in individuals with upper limb musculoskeletal disorders [40]. As a result, the ULFI-PL may be a valuable tool for both clinicians and researchers in monitoring patient improvements and guiding rehabilitation strategies. Our result is comparable to the original version, where a large degree of responsiveness was also confirmed using ES = 0.93 and SRM = 1.33 [14]. The ULFI-Ar [25] and ULFI-Gr [26] obtained a moderate degree of responsiveness (ES = 0.67, SRM = 0.667 and ES = 1.19, SRM = 1.31, respectively), and the ULFI-FC obtained a moderate to large degree of responsiveness (ES = 0.62; SRM = 0.88) [17]. Our study also demonstrated a moderate and statistically significant correlation between changes in the ULFI-PL and QuickDASH-PL, confirming the ability of the ULFI-PL to detect changes in patients' conditions. Similar results were obtained for the French-Canadian version, where the external responsiveness showed a moderate and statistically significant correlation between the change score of the ULFI-FC and DASH-FC (*r* = -0.64) [17]. Other versions did not assess this psychometric property of the ULFI. In this study, no floor or ceiling effects were observed, indicating that the items of the ULFI are composed within an optimal range to detect any changes. In therapeutic contexts, if participants are already scoring at or near the maximum, it becomes difficult to measure an improvement or the effectiveness of an intervention, as there is no "space" to demonstrate further progress [31]. No floor or ceiling effects were observed in the original ULFI [14] or in the Persian [22], Brazilian Portuguese [21], Korean [20], French-Canadian [16], or Arabic [24] language versions. Researchers from Turkey [18], Spain [15], and Italy [19] did not report information on this case. The MCID was calculated for the ULFI-PL between Test 1 and Test 3 (eight-week interval with physiotherapy applied) using the distribution-based method, and it amounted to 10.41 points (95%CI: 7.62–16.41) and 13.06% (95% CI:9.52–19.75). This means that an improvement of at least 10.41 points is considered the minimal clinically meaningful change for patients, with 95% confidence that the true change in the patient's condition lies between 7.62 and 16.41 points. The ULFI-PL effectively detects changes in a disability following physiotherapy in patients with ULMSDs, making it a valid primary endpoint for clinical trials. The first and only study that previously presented the MCID for the ULFI in patients with ULMSDs was conducted by Greek researchers. It was calculated using an ROC curve analysis, which indicated that a substantial improvement was defined as a change of 26% or greater in the total ULFI-Gr score [26].

The practical characteristics, such as clarity and comprehension, the burden through completion and scoring, and the properties of missing responses are also important for measurement tools. Those with a low practicality are less likely to gain acceptance from clinicians and be incorporated into everyday clinical practice. The original ULFI is characterized by its understandability, brevity, easy transferability to a 100-point scale, and rapidity of completion and scoring [10, 14]. The ULFI-PL also demonstrated very good practical characteristics, with a high understandability of questions, and no missing responses detected in the group of 127 participants, a short completion time ($\overline{x}$ = 144 ± 40 s; original ULFI, $\overline{x}$ = 117 ± 47 s), and a scoring time of $\overline{x}$ = 18 ± 6 s vs. $\overline{x}$ = 16 ± 4 s.

### Limitations and strengths

A limitation of the current study is that all the participants were recruited from the same hospital, which may affect the generalizability of the results. However, our sample is gender-representative of the Polish population. According to data from the Central Statistical Office in Poland (GUS) for 2023, women constitute 51.6% of the population, which is comparable to the studied group (53.5%). Our sample is slightly older ($\overline{x}$ = 45.07 years, Me = 47) compared to the general Polish population ($\overline{x}$ = 42.6 years, Me = 42). This difference may be attributed to the nature of the studied group, which consists of individuals with injuries or musculoskeletal disorders requiring physiotherapy (https://stat.gov.pl/obszary-tematyczne/ludnosc/ludnosc/ludnosc-stan-i-struktura-ludnosci-oraz-ruch-naturalny-w-przekroju-terytorialnym-w-2023-r-stan-w-dniu-31-12%2C6%2C36.html?pdf=1&utm_source=chatgpt.com.).

Additionally, while the sample size was adequate for the CFA according to the COSMIN recommendations, a minimum of 175 patients would be necessary to achieve a very good sample size as per these guidelines, which would definitively clarify the factor structure through the CFA [42]. Furthermore, a larger sample could improve generalizability by enabling more robust subgroup analyses and a more detailed assessment of the ULFI-PL’s performance across specific conditions. It could also help mitigate potential biases related to variability in patient characteristics. Nevertheless, our sample size aligns with established COSMIN guidelines for PROMs validation studies and provides sufficient statistical power for psychometric analysis [42].

A notable strength of this study is the comprehensive evaluation of all major psychometric properties—reliability, validity, and responsiveness, as well as the practical characteristics of the ULFI-PL questionnaire. Our study, alongside the findings of researchers from Greece [26], is the second published study that enhances the interpretability of the ULFI-PL by utilizing the MCID, which will aid clinicians in quickly assessing patient improvements following physiotherapy. Nevertheless, the MCID should be interpreted with care and caution because this feature is context-specific in its use.

Additional strengths include the prospective design and the variety of ULMSDs affecting each upper limb sub-region, with different severity levels. The recruited participants had acute, subacute, or chronic conditions, allowing for the assessment of the applicability of the ULFI-PL at every stage of a disease or injury, including post-surgery. Moreover, the absence of a marked ceiling effect in the results of our research is crucial for ensuring the instrument's ability to detect changes and variations across all functional levels of the upper limb, making it suitable for use in a broader clinical context.

In summary, the ULFI-PL, as a short and simple PROM, can be used by clinicians to assess upper limb function impairment caused by musculoskeletal disorders. Its responsiveness, as demonstrated by our findings, suggests that it is a valuable tool in rehabilitation settings, aiding in treatment decisions and therapy adjustments based on patient-reported outcomes.

### Future considerations

The responsiveness and the MCID of a PROM are not constant features, but they depend on the characteristics of the population, the severity and chronicity of the condition, the type of intervention, and the duration of the follow-up period [25]. Thus, future research on the questionnaire should focus on validating the ULFI-PL in various settings, including different hospitals and among patients receiving treatments other than physiotherapy. A larger sample size should be used to assess its responsiveness and interpretability separately for each patient group with different conditions.

## Conclusions

Based on our findings, the Polish version of the ULFI has a unidimensional structure, an adequate internal consistency and construct validity, an excellent test–retest reliability, and a high degree of responsiveness following physiotherapy. The ULFI-PL also demonstrates very good practical characteristics, as it is fully understandable, concise, and quick to complete and score. This indicates that the ULFI-PL is suitable for use as the outcome measure in clinical and research settings for Polish-speaking patients with upper limb musculoskeletal disorders.

## Data Availability

The data that support the findings of this study are available from the corresponding author upon reasonable request.

## Abbreviations

*CFA*: Confirmatory factor analysis
*CFI*: Comparative fit index
*df*: Degrees of freedom
*EMA*: European Medicines Agency
*ES*: Effect size
*FDA*: Food and Drug Administration
*GRC*: Global Rating of Change scale
*ICC*: Intraclass correlation
*ISPOR*: International Society for Pharmacoeconomics and Outcomes Research
*MCID*: Minimal clinically important difference
*MDC*: Minimal detectable change
*Me*: Median
*MSDs*: Musculoskeletal disorders
*n*: Number
*NPRS*: Numeric Pain Rating Scale
*p*: Statistical significance
*PROMs*: Patient-reported outcome measures
*QuickDASH-PL*: Shortened Disabilities of the Arm, Shoulder, and Hand questionnaire—Polish version
*RMSEA*: Root mean square error of approximation
*r*: Pearson’s correlation coefficient
*SD*: Standard deviation
*SEM*: Standard error of measurement
*SFI*: Spine Functional Index
*SPADI*: Shoulder Pain and Disabilities Index
*SRM*: Standardized response mean
*SRMR*: Standardized root mean square residual
*TLI*: Tucker–Lewis index
*UEFS*: Upper Extremities Functional Scale
*ULFI*: Upper Limb Functional Index
*ULFI-Br*: Upper Limb Functional Index—Brazilian Portuguese version
*ULFI-FC*: Upper Limb Functional Index—French-Canadian
*ULFI-Ar*: Upper Limb Functional Index—Arabic version
*ULFI-Gr*: Upper Limb Functional Index—Greek version
*ULFI-I*: Upper Limb Functional Index—Italian version
*ULFI-K*: Upper Limb Functional Index—Korean version
*ULFI-PL*: Upper Limb Functional Index—Polish version
*ULFI-Pr*: Upper Limb Functional Index—Persian version
*ULFI-Sp*: Upper Limb Functional Index—Spanish version
*ULFI-Tr*: Upper Limb Functional Index—Turkish version
*ULFI-U*: Upper Limb Functional Index—Urdu version
*ULs*: Upper limbs
*WHOQOL-BREF-PL*: Polish version of the World Health Organization Quality of Life—BREF
*WORC*: Western Ontario Rotator Cuff questionnaire
%: Percent
$$\overline{x}$$: Mean
*χ*^*2*^: Chi-squared
